# Associations of four important dietary pattern scores, micronutrients with sarcopenia and osteopenia in adults: results from the National Health and Nutrition Examination Survey

**DOI:** 10.3389/fnut.2025.1583795

**Published:** 2025-07-23

**Authors:** Yuan Li, Wen Gu, Wen Hao, Yadan Xu, Kexin Li, Yueliang Zhao, Qingyun Huang

**Affiliations:** ^1^Department of Orthopaedics, Peace Hospital of Changzhi Medical College, Changzhi, China; ^2^State Key Laboratory of Systems Medicine for Cancer, Center for Single-Cell Omics, School of Public Health, Shanghai Jiao Tong University School of Medicine, Shanghai, China; ^3^Qingdao Municipal Center for Disease Control and Prevention, Qingdao, China; ^4^Qingdao Institute of Preventive Medicine, Qingdao, China

**Keywords:** dietary patterns, micronutrients, sarcopenia, osteopenia, NHANES

## Abstract

**Introduction:**

We aimed to comprehensively evaluate the association between four important dietary patterns and micronutrients with sarcopenia and osteopenia. Data were obtained from the National Health and Nutrition Examination Survey 2005-2018.

**Methods:**

The association between dietary patterns and micronutrients with sarcopenia and osteopenia was evaluated by logistic regression models. 6709 and 7161 participants were included in the final analyses.

**Results:**

Higher adherence to HEI-2020, aMed, and DASH dietary patterns was inversely associated with sarcopenia risk, with each standard deviation (SD) increment linked to 18%, 16%, and 14% reductions in odds, respectively. However, DII increased the prevalence of sarcopenia and osteopenia by 44% and 8% per SD increase. Dietary intake of vitamins A, B2, B9, C, calcium, phosphorus, magnesium, copper, and potassium were negatively associated with sarcopenia, whereas vitamins D and K were negatively associated with osteopenia. SIRI and NLR partially mediated the associations among them.

**Discussion:**

Adherence to healthy dietary patterns especially anti-inflammatory diet and supplementary micronutrients reduce bone and muscle loss.

## 1 Introduction

The impact of sarcopenia on the human body is increasingly being explored. It affects 10 to 16% of the world’s elderly with a higher prevalence in some metabolic diseases such as diabetes (1). Sarcopenia is closely related to fracture, metabolic syndrome, diabetes, hypertension and other adverse events (1–3). Recently, diets are considered to be interfere with sarcopenia by regulating metabolism for diets are closely related to energy metabolism, lipid metabolism and systemic inflammation (4, 5). Many studies have confirmed the function of protein, but the latest results show that in the elderly population, the effect of protein supplement alone may not be significant (6). That means the fine regulation effect of micronutrients such as vitamin D, calcium and phosphorus is increasingly prominent (7, 8), and it triggered thinking about the interaction between different nutrients (9). The diet model of studying the overall relationship between nutrients and metabolic system seems to have more practical value.

At the first critical point of bone mineral density decline, osteopenia monitoring is of great value. Similar to sarcopenia, osteopenia is also a disease closely related to diet. Studies have confirmed the negative correlation between the nutritional mode characterized by protein, polyunsaturated fatty acids and micronutrients and osteopenia (10), especially the diets represented by calcium and vitamin D (11). In addition, dietary bioactive substances, such as vitamin K, and antioxidants, such as retinol, have also been involved in many studies (12, 13).

As important diseases of the musculoskeletal system, osteopenia and sarcopenia show significant common variation in many physiological and pathological mechanisms (4). Studies have pointed out the significant harm of the coexistence of these two diseases, especially the synergistic effect on adverse outcomes such as malnutrition, falls, fractures and death (14–16). For the treatment of osteopenia and sarcopenia, consensus is that resistance training combined with diet intervention is the best measure (17), emphasizing the diet model represented by protein and calcium (6, 18). In the further study of the combined mechanism, a series of metabolic disorders such as steatosis, chronic inflammation, insulin resistance, and decreased secretion of growth hormone have opened up a new micro vision for us (4, 19, 20). Therefore, the combination of sarcopenia and osteopenia is of great significance to understand the internal physiology of the musculoskeletal system as a whole.

The Healthy Eating Index-2020 (HEI-2020), a cornerstone for evaluating adherence to U.S. dietary guidelines, assesses nutritional density (e.g., per 1,000 kcal) rather than mere energy intake (21, 22). Emerging evidence links higher HEI scores to better muscle-related outcomes, including grip strength, underscoring the role of early-life nutrition in optimizing midlife muscle function and attenuating age-related sarcopenia progression (23). Notably, HEI also demonstrates protective effects against osteopenia (11). Conversely, the Dietary Inflammatory Index (DII)—which quantifies diet-driven inflammation—correlates with musculoskeletal decline and metabolic disorders (24). The alternate Mediterranean (aMed) diet, rich in n-3 fatty acids and antioxidants, shows 10–18% lower nutrient deficiencies in sarcopenic patients, though associations with osteopenia remain underexplored (25). The DASH diet presents conflicting evidence: while one study found no significant sarcopenia link in older adults (26), another reported reduced myostatin levels (a muscle atrophy biomarker) with DASH adherence (27). For osteopenia, preliminary data suggest DASH may enhance lumbar bone mineral density, though larger cohorts are needed for confirmation (28). These findings collectively highlight dietary patterns’ differential impacts on musculoskeletal aging.

This paper used the data from the National Health and Nutrition Examination Survey (NHANES) to investigate the association of four healthy dietary patterns and micronutrients with sarcopenia and osteopenia. This study aimed to explore the correlation between nutrition and motor system diseases and further analyze the common mechanism of dietary patterns.

## 2 Materials and methods

### 2.1 Study population and design

This study used population data collected by NHANES from 2005 to 2018, with a total of 76,496 participants initially. After excluding those without data of dietary intake, inflammatory index and body mass index (BMI), 32,468 participants had exposure data. To reduce bias, people without information of diabetes history (*n* = 19), pregnant women (*n* = 234), and people without relevant covariate data (*n* = 2,426) were excluded. At the same time, in order to avoid errors caused by musculoskeletal growth and development, participants younger than 18 years old (*n* = 9,800) were also excluded from this study. The final study included 6,709 participants had appendicular bone mass data on sarcopenia and 7,161 participants had bone mineral density data on osteopenia (Supplementary Figure S1). All participants provided written informed consent. The NHANES protocol was approved by the Centers for Disease Control (CDC) and the prevention institutional review board.

### 2.2 Dietary scores

NHANES uses 24-h recall data to assess dietary intake. Professionals collect information about the type and quantity of dietary intake of participants in the past 24 h through interviews, and use this as a basis to evaluate the energy, nutrients and other food ingredients provided by these foods and beverages. Whether they are having a certain type of special diet will also be asked. The follow-up meeting was held after 3–10 days, and the interviewer will call all participants back. If dietary information was available on both occasions, the average value was taken. Data from dietary interview questionnaires and food pattern equivalent database files were used to calculate dietary scores (29), and all nutrients related to dietary patterns were substituted as exposures for analysis.

#### 2.2.1 Healthy eating index-2020

The healthy eating index (HEI) was jointly developed by the United States Department of Agriculture (USDA) and the National Cancer Institute (NCI), and its initial purpose was to evaluate the implementation of Dietary Guidelines for Americans (DGA) (30). HEI is composed of 13 independent items representing different food categories and nutrients. These 13 items are further classified into adequacy components and moderation components. Adequacy components such as fruits and vegetables are encouraged to be consumed, and the higher the intake, the higher the score, while moderation components such as saturated fat is restricted, and the more the intake, the lower the score. The maximum score of each item is 5 or 10, and the total score is 100. The higher the score, the better the dietary compliance (22). HEI-2020 is the latest version revised in 2020, maintaining the consistency with the DGA from 2020 to 2025 (Supplementary Table S1).

#### 2.2.2 Dietary inflammatory index

DII is a new tool for exploring the contribution of various dietary components to inflammation. It is divided into anti-inflammatory or pro-inflammatory properties based on 45 parameters of nutrients, compounds and foods. Among them, there are 28 food parameters available for calculation in NHANES (Supplementary Table S1). In this paper, DII adjusted by energy intake was calculated according to the food intake per 1,000 kcal of energy intake. The total DII value is the sum of each DII value. The higher the positive DII value, the greater the contribution to inflammation, and the higher the negative DII value, the greater the contribution to anti inflammation (31).

#### 2.2.3 Alternative Mediterranean diet score

aMed was constructed from a total of nine food components as an index (Supplementary Table S1) to assess adherence to the Mediterranean dietary pattern. Women in the median level of the whole cohort were scored as “0,” for beneficial dietary components, a score equal to or higher than this level was 1, a score lower than this level was 0, and 0 was regarded as a harmful factor. For dairy and meat products and moderate alcohol consumption, the scoring rules are opposite, that is, score 0 at or above this level, and score 1 below this level. The total score of aMed is the sum of the scores of 9 items, and the value is between 0 and 9. The higher the score, the better the compliance of aMed mode (32).

#### 2.2.4 Dietary approaches to stop hypertension

DASH consists of nine selected food components (Supplementary Table S1), which are used to evaluate the compliance of the dietary pattern conducive to reducing blood pressure. The food pattern equivalence database (FPED) was used to convert nutritional data into food groups to calculate various dietary indexes. Each item meets the goal and gets 1 point. The total score of DASH ranges from 0 to 9. The higher the score, the better the compliance (33).

### 2.3 Micronutrients

All participants provided initial 24-h dietary recall data collected via a validated Food Frequency Questionnaire (FFQ), ensuring high data precision (11). Over 80% of the cohort completed two independent 24-h dietary recalls, enhancing measurement reliability through replication. For the remaining participants who contributed only a single recall, the initial dataset retained robust methodological integrity, as it was systematically validated against energy intake plausibility criteria, nutrient distribution patterns, and exclusion of implausible reporters. VA, VB1, VB2, VB3, VB6, VB9, VB12, VC, VD and VK were vitamins included in the analysis. The study also included minerals needed by the human body, including calcium, phosphorus, magnesium, iron, zinc, copper, sodium, potassium and selenium. All micronutrient intakes were analyzed in triplicate.

### 2.4 Inflammatory index assessment

We obtained the results of routine blood tests for each participant. Blood was collected from participants aged 1 year and older by a phlebotomist. The amount of blood drawn varied by age. Blood was processed and aliquoted into vials. The vials were then refrigerated or frozen before transport to laboratories across the United States. Except for complete blood count (CBC) and pregnancy tests, which were performed in the MEC, most assays were completed in 35 laboratories across the United States. If consent was granted, some specimens were stored for future studies. Blood was processed and aliquoted into vials. The vials were then refrigerated or frozen before transport to laboratories. We calculated the Neutrophil-to-Lymphocyte Ratio (NLR) and Systemic Inflammation Response Index (SIRI) according to the following equations: NLR = neutrophil count/lymphocyte count; SIRI = (neutrophil count × monocyte count)/lymphocyte count (34).

### 2.5 The diagnosis of the appendicular skeletal muscle mass index, total femur BMD, femoral neck BMD and lumbar spine BMD

ASMI was calculated by appendiceal skeletal muscle mass ASMM divided by the square of height (35). BMD represented for bone mineral density. Total femur BMD, femoral neck BMD and lumbar spine BMD were parts of the definition of osteopenia as indicated below.

### 2.6 The diagnosis of sarcopenia, osteopenia, and osteoporosis

Combined with the definition of sarcopenia in several studies, this study finally defined sarcopenia as a sex-specific appendicular skeletal muscle mass (ASM) value less than the normal value minus 2.0 standard deviations (36). In NHANES, ASM was determined by summing the lean muscle mass of the arms and legs, as measured by dual-energy X-ray absorptiometry (DXA). After undergoing BMD testing by dual-energy X-ray absorptiometry (DXA) examinations, participants were divided into three groups (normal, osteopenia, and osteoporosis). Individuals with BMD score of 2.5 standard deviations or more below the normal value were considered osteoporosis, individuals with BMD score between 2.5 lower and 1.0 lower standard deviations from normal values were considered osteopenia (37).

### 2.7 Covariates

The regression model was adjusted for age, sex, race, body mass index, poverty status, education level, history of diabetes, smoking status, alcohol consumption, leisure time physical activity and total energy intake. Among them, age, body mass index, alcohol consumption and and total energy intake were continuous variables, sex, race, poverty status, education level, history of diabetes, smoking status and leisure time physical activity was a categorical variable in NHANES. Poverty status was evaluated by the income-to-population ratio (PIR).

### 2.8 Statistical analysis

The overall characteristics of participants were stratified according to whether sarcopenia and osteopenia were confirmed. Considering the complex sampling characteristics and country representativeness of NHANES, all analyses were weighted. Logistic regression was used to evaluate the association between four important dietary pattern scores, micronutrients, and sarcopenia and osteopenia. Dietary pattern scores and micronutrients were classified according to tertiles (T1, T2, T3). In the regression model, model 1 adjusted for gender (male and female), age (continuous, year), race (non Hispanic White, Hispanic, etc.), body mass index (continuous, kg/m2), poverty income ratio (PIR) (<1.3, 1.3–3.5, ≥ 3.5), education level (under high school and high school or above), and history of diabetes (yes and no). On the basis of model 1, model 2 also adjusted for smoking status (never smoking and smoking), alcohol consumption (continuous, g/day), and leisure time physical exercise (LTPA) (sufficient and insufficient) (38). In addition, in order to reduce the bias caused by dietary intake differences between individuals, the total energy intake was also adjusted to maintain balance. Based on the results of multivariable logistic regression, the dose–response relationship among dietary pattern score, micronutrients, sarcopenia and osteopenia was explored using the restricted cubic spline (RCS) to explore the non-linear relationship between various exposure indicators and sarcopenia and osteopenia, and three nodes were set up in the model. Risk estimates were adjusted for covariates consistent with model 2. In the model, the nonlinearity is estimated according to the *p*-values. Statistical significance was considered as a *p*-value < 0.05, and all tests were two tailed. All statistical analyses were performed using R (4.2.3). The calculation of the dietary pattern scores were performed using the “Dietaryindex” package.

## 3 Results

### 3.1 Population characteristics

We included 6,709 and 7,161 participants from NHANES 2005–2018, of whom 738 (11.0%) and 2,947 (41.2%) were diagnosed with sarcopenia and osteopenia, respectively (Table 1). Participants with sarcopenia had less LTPA (32.7%), lower BMI (21.0 ± 2.5) and lower ASMI (5.9 ± 0.9) than those without sarcopenia. Participants with osteopenia had older age (54.3 ± 15.8), lower PIR (2.5 ± 1.6), lower educational level (26.6%), less LTPA (30.5%), higher proportion of smokers (46.0%), lower BMI (26.6 ± 5.2) than those without osteopenia. Gender and ethnicity were significantly different in the osteopenia subgroup, but only ethnicity was significantly different in the sarcopenia subgroup (*p*-value < 0.001). Supplementary Table S2 shows the dietary intake of vitamins and minerals in each group and the differences between groups.

**Table 1:** Baseline characteristics of participants in the NHANES.

Characteristics	Sarcopenia			Osteopenia		
	Yes (*N* = 738)	No (*N* = 5,971)	*p*-value	Yes (*N* = 2,947)	No (*N* = 4,214)	*p*-value
Age, mean ± SD, years	36.2 ± 13.2	38.5 ± 12.0	<0.001	54.3 ± 15.8	44.3 ± 14.7	<0.001
Gender, *n* (%)			0.463			<0.001
Male	348 (47.2)	2,901 (48.6)		1,173 (39.8)	2,254 (53.5)	
Female	390 (52.9)	3,070 (51.4)		1774 (60.2)	1960 (46.5)	
Ethnicity, *n* (%)			<0.001			<0.001
Mexican American	81 (11.0)	895 (15.0)		539 (18.3)	721 (17.1)	
Other Hispanic	64 (8.7)	608 (10.2)		325 (11.0)	401 (9.5)	
Non-Hispanic White	293 (39.7)	2,231 (37.4)		1,525 (51.8)	1919 (45.5)	
Non-Hispanic Black	52 (7.1)	1,364 (22.8)		337 (11.4)	933 (22.1)	
Other Race-Including Multi-Racial	248 (33.6)	873 (14.6)		221 (7.5)	240 (5.7)	
Educational level, *n* (%)			0.133			<0.001
Less than high school	114 (15.5)	1,055 (17.7)		785 (26.6)	964 (22.9)	
High school or above	624 (84.6)	4,916 (82.3)		2,162 (73.4)	3,250 (77.1)	
Poverty-income ratio, mean ± SD	2.5 ± 1.7	2.5 ± 1.7	0.855	2.5 ± 1.6	2.6 ± 1.6	0.010
Leisure time physical activity, *n* (%)			<0.001			<0.001
Adequate	241 (32.7)	2,494 (41.8)		898 (30.5)	1,575 (37.4)	
Inadequate	497 (67.3)	3,477 (58.2)		2049 (69.5)	2,639 (62.6)	
Smoking status, *n* (%)			0.482			0.030
Smoking	270 (36.6)	2,264 (37.9)		1,354 (46.0)	1827 (43.4)	
Non-smoking	468 (63.4)	3,707 (62.1)		1,593 (54.1)	2,387 (56.6)	
Alcohol consumption, mean ± SD, g/day	7.4 ± 21.5	8.7 ± 21.5	0.120	6.6 ± 16.4	9.8 ± 22.9	<0.001
History of diabetes, *n* (%)			0.001			0.051
Yes	30 (4.1)	451 (7.6)		328 (11.1)	409 (9.7)	
No	708 (95.9)	5,520 (92.5)		2,619 (88.9)	3,805 (90.3)	
BMI, mean ± SD, kg/m^2^	21.0 ± 2.5	29.8 ± 6.7	<0.001	26.6 ± 5.2	29.4 ± 5.8	<0.001
ASMI, mean ± SD, kg/m^2^	5.9 ± 0.9	8.3 ± 1.6	<0.001	–	–	–
Total femur, mean ± SD, g/cm^2^	–	–	–	0.8 ± 0.1	1.1 ± 0.1	<0.001
Femoral Neck, mean ± SD, g/cm^2^	–	–	–	0.7 ± 0.1	0.9 ± 0.1	<0.001
Lumbar Spine, mean ± SD, g/cm^2^	–	–	–	0.9 ± 0.1	1.1 ± 0.1	<0.001

Descriptive data were shown as mean (SD) while categorical variables were reported as *n* (%). *p* value < 0.05 were considered significant. –: data not available. NHANES, National Health and Nutrition Examination Survey; SD, standard deviation; BMI, body mass index; ASMI, Appendicular Skeletal Mass Index.

### 3.2 Association between dietary scores, micronutrients and sarcopenia, osteopenia and osteoporosis

**Figure 1:**
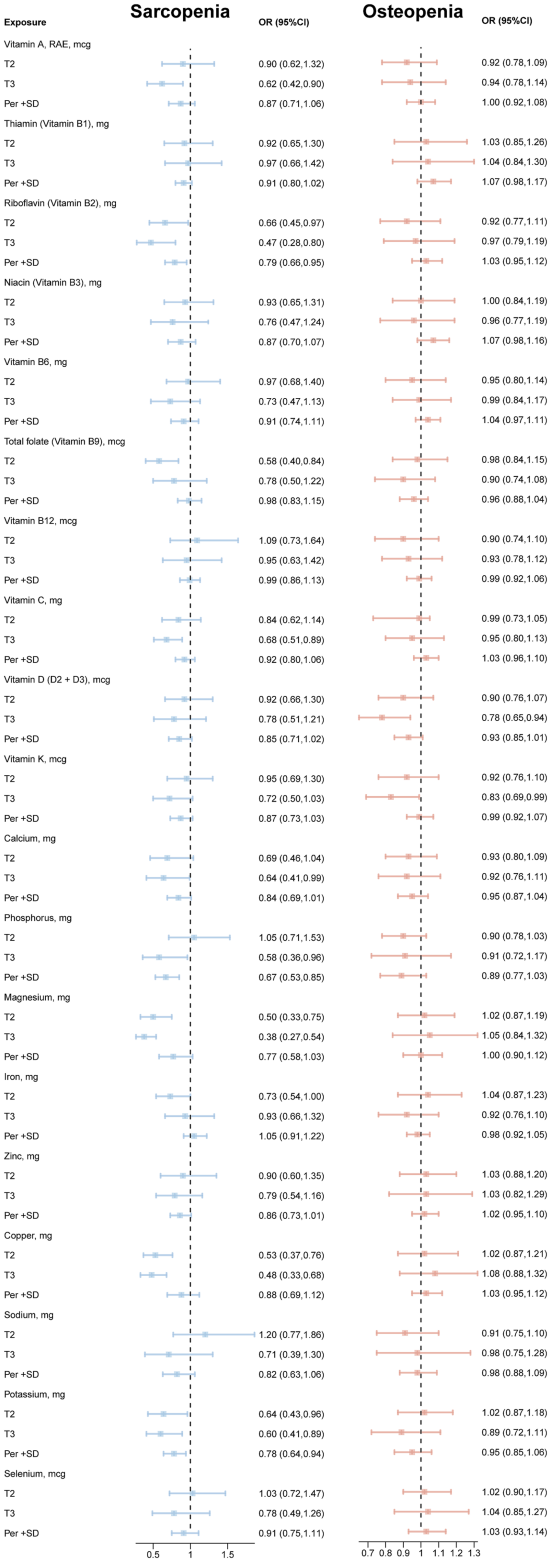
Associations between vitamins, minerals and sarcopenia and osteopenia. Model: age, sex, race/ethnicity, body mass index, poverty status, education level, history of diabetes, smoking status, alcohol consumption, leisure time physical activity and total energy intake.

As shown in the figure (Table 2), in both models, HEI-2020 score was significantly negatively correlated with sarcopenia (*p*-value = 0.002, *p*-value = 0.048), significantly reducing the incidence of sarcopenia by 35%, while DII was significantly positively correlated with sarcopenia (*p*-value < 0.001, *p*-value = 0.003), significantly increasing the incidence of sarcopenia by 77%. aMed was significantly negatively correlated with sarcopenia only in model 1 (*p*-value = 0.015). For each SD increase in the three dietary pattern scores, HEI2020, AMED, and DASH, the prevalence of sarcopenia decreased by 18, 16, and 14%, respectively. In addition, for each SD increase in DII, the prevalence of sarcopenia and osteopenia increased by 44 and 8%, respectively. As shown in Figure 1, micronutrients VB2, magnesium, copper and potassium were significantly negatively correlated with sarcopenia at T2 or T3 intake, VB9 was significantly negatively correlated with sarcopenia only at T2 intake, and VA, VC, calcium and phosphorus were significantly negatively correlated with sarcopenia only at T3 intake. VD and VK were significantly negatively correlated with osteopenia only at T3 intake [OR (95%CI): 0.78 (0.65, 0.94); OR (95%CI): 0.83 (0.69, 0.99)].

**Table 2:** Analysis of the association between dietary patterns and sarcopenia and osteopenia.

Dietary patterns	Sarcopenia						Osteopenia					
	Model 1			Model 2			Model 1			Model 2		
	OR	95%CI	*p*-value	OR	95%CI	*p*-value	OR	95%CI	*p*-value	OR	95%CI	*p*-value
HEI-2020												
T1	Reference			Reference			Reference			Reference		
T2	0.76	(0.55, 1.05)	0.093	0.83	(0.59, 1.18)	0.290	1.06	(0.92, 1.22)	0.409	1.11	(0.97, 1.27)	0.132
T3	0.52	(0.35, 0.78)	0.002	0.65	(0.43, 1.00)	0.048	0.88	(0.73, 1.07)	0.205	0.96	(0.79, 1.16)	0.667
Per + SD	0.82	(0.71, 0.95)	0.011	0.92	(0.79, 1.08)	0.324	0.98	(0.90, 1.06)	0.546	1.02	(0.94, 1.10)	0.699
DII												
T1	Reference			Reference			Reference			Reference		
T2	1.50	(1.01, 2.23)	0.044	1.33	(0.92, 1.94)	0.111	1.15	(0.95, 1.38)	0.155	1.11	(0.92, 1.33)	0.286
T3	2.17	(1.67, 2.82)	<0.001	1.77	(1.35, 2.32)	0.003	1.08	(0.95, 1.23)	0.227	1.01	(0.89, 1.14)	0.931
Per + SD	1.44	(1.29, 1.60)	<0.001	1.32	(1.17, 1.48)	<0.001	1.08	(1.01, 1.14)	0.020	1.02	(0.97, 1.08)	0.418
aMed												
T1	Reference			Reference			Reference			Reference		
T2	0.70	(0.49, 1.01)	0.054	0.69	(0.47, 0.99)	0.046	0.93	(0.79, 1.10)	0.377	0.95	(0.81, 1.11)	0.493
T3	0.62	(0.42, 0.91)	0.015	0.73	(0.49, 1.09)	0.124	0.89	(0.75, 1.06)	0.184	0.93	(0.79, 1.10)	0.407
Per + SD	0.84	(0.72, 0.98)	0.029	0.92	(0.79, 1.08)	0.309	0.97	(0.90, 1.05)	0.426	0.99	(0.93, 1.07)	0.856
DASH												
T1	Reference			Reference			Reference			Reference		
T2	0.94	(0.66, 1.34)	0.731	0.97	(0.67, 1.40)	0.882	0.98	(0.82, 1.17)	0.847	1.03	(0.86, 1.22)	0.775
T3	0.76	(0.52, 1.11)	0.151	0.93	(0.62, 1.38)	0.710	0.91	(0.74, 1.13)	0.386	0.97	(0.78, 1.20)	0.759
Per + SD	0.86	(0.75, 0.98)	0.025	0.97	(0.84, 1.13)	0.689	1.00	(0.92, 1.08)	0.946	1.02	(0.94, 1.11)	0.568

Model 1: Adjusted for age, sex, race/ethnicity, body mass index, poverty status, education level, history of diabetes; Model 2: Model 1 + Smoking status + Alcohol consumption + Leisure time physical activity + Total energy intake. *p*-values less than 0.05 (*p*-value <0.05) were considered significant. OR, odd ratio; CI, confidence interval; HEI, healthy eating index; DII, Dietary Inflammation Index; aMed, Alternate Mediterranean Diet Score; DASH, Dietary Approaches to Stop Hypertension Index.

The results showed that (Supplementary Figure S2), in both models, no significant association was found between the four dietary patterns and osteoporosis. VC and VK were significantly negatively correlated with osteoporosis at T2 or T3 intake, while VB1, VB12, VD, phosphorus, iron, sodium and potassium were significantly negatively correlated with osteoporosis only at T3 intake.

### 3.3 Subgroup analysis of gender

**Figure 2:**
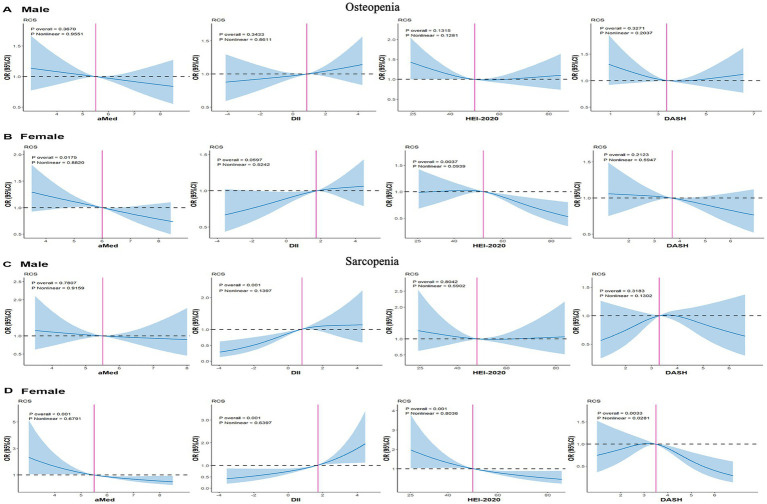
RCS plots of the association of dietary scores with sarcopenia and osteopenia in gender subgroups. **(A)** osteopenia-male. **(B)** osteopenia-female. **(C)** sarcopenia-male. **(D)** sarcopenia-female. Model: age, sex, race/ethnicity, body mass index, poverty status, education level, history of diabetes, smoking status, alcohol consumption, leisure time physical activity and total energy intake. HEI, healthy eating index; DII, dietary inflammation index; aMed, alternate Mediterranean diet; DASH, dietary approaches to stop hypertension.

As shown in Figure 2, in females, DASH showed a significant barbed curve association with sarcopenia, and the other three dietary scores did not show a significant nonlinear relationship with sarcopenia and osteopenia in different genders.

### 3.4 Analysis of the association between DII and sarcopenia in the subgroup of osteopenia

As shown in the figure (Supplementary Table S3), there was a significant multiplicative interaction between DII and osteopenia on sarcopenia (*p*-value = 0.037). The results showed that in model 1 and model 2, the DII score showed a significant association with sarcopenia in the osteopenia subgroup (*p*-value = 0.021, *p*-value = 0.024), while it was not significantly in the non-osteopenia subgroup.

### 3.5 Mediating role of inflammatory index

In order to explore the mediating effects of SIRI and NLR between dietary patterns and indicators of sarcopenia and osteopenia, four dietary scores were used as independent variables, ASMI, total femur BMD, femoral neck BMD, lumbar spine BMD as dependent variables, SIRI and NLR as mediating variables, and logistic regression analysis was performed.

**Figure 3:**
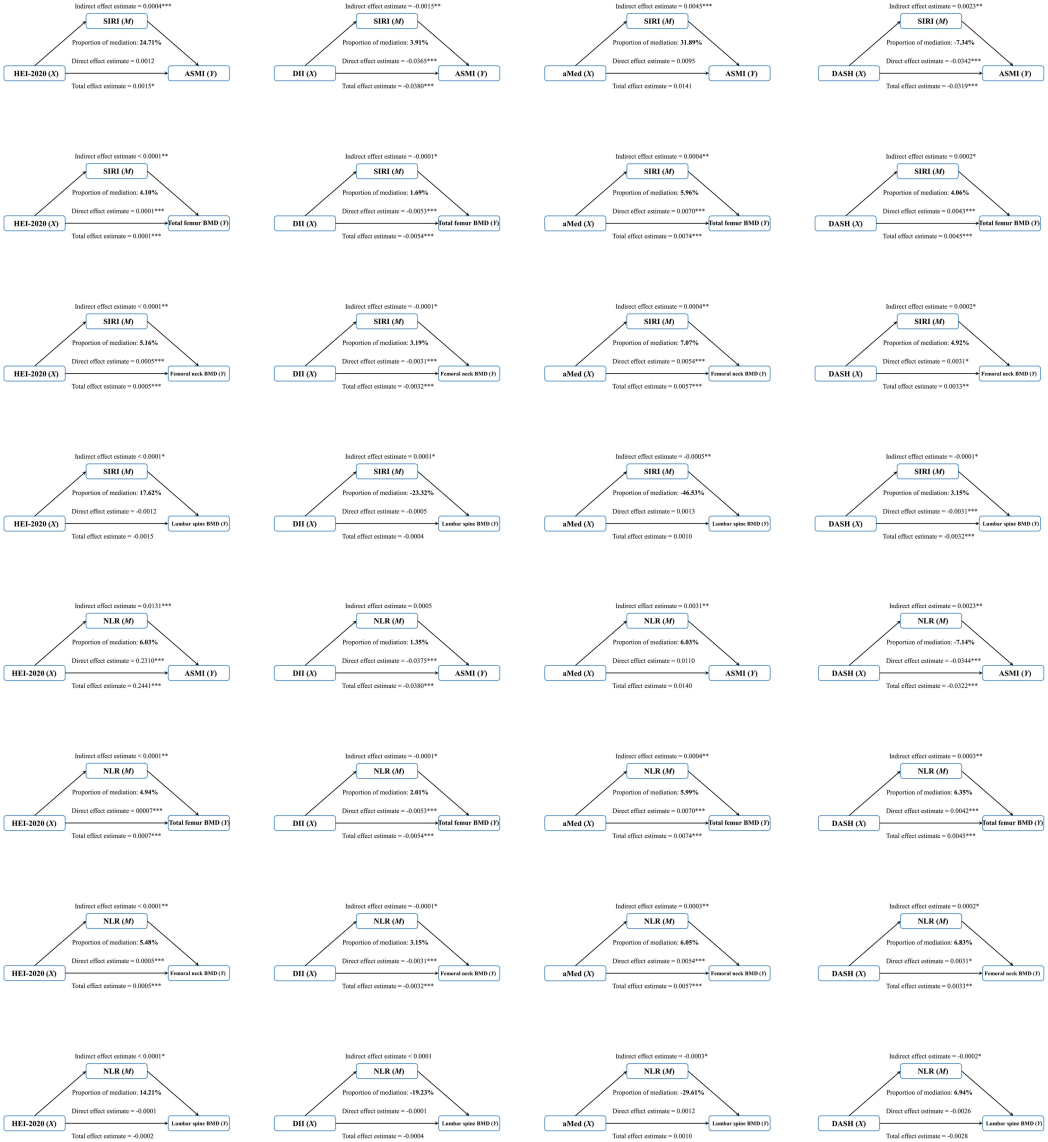
Inflammation index’s mediate the association between dietary patterns and sarcopenia and osteopenia. Model: age, sex, race/ethnicity, body mass index, poverty status, education level, history of diabetes, smoking status, alcohol consumption, leisure time physical activity and total energy intake.

Figure 3 shows that SIRI plays a positive partial mediating effect in the association of four dietary patterns with total femur BMD and femoral neck BMD (HEI-2020: proportion = 4.1%, proportion = 5.2%; DII: proportion = 1.7%, proportion = 3.2%; aMed: proportion = 6.0%, proportion = 7.1%; DASH: proportion = 4.1%, proportion = 4.9%). Similarly, NLR also plays a positive partial mediating effect in the association of four dietary patterns with total femur BMD and femoral neck BMD (HEI-2020: proportion = 4.9%, proportion = 5.5%; DII: proportion = 2.0%, proportion = 3.2%; aMed: proportion = 6.0%, proportion = 6.1%; DASH: proportion = 6.4%, proportion = 6.8%). In addition, SIRI partially mediated the association between DII and ASMI (proportion = 3.9%). NLR partially mediated the association between HEI-2020 and ASMI (proportion = 6.0%). Only SIRI mediated the association between DASH and lumbar spine BMD (proportion = 3.2%).

## 4 Discussion

This is the first comprehensive assessment of the association between four important dietary patterns, micronutrients, and sarcopenia and osteopenia using a nationally representative NHANES cohort, and to explore possible inflammatory pathways. In NHANES, we found that the HEI-2020, aMed and DASH scores were significantly negatively correlated with the occurrence of sarcopenia, which are consistent with previous research results as follows. A study on the elderly in Iran showed that the average HEI score of the possible sarcopenia group was 3.67 points lower than normal group (39). Our study extended the population to adults older than 18 years and adopted logistic regression to clarify the negative association between HEI score and sarcopenia. aMed is characterized by plant diet and polyunsaturated fatty acids (PUFA) (40). An intervention study of PUFA combined with resistance exercise observed that type IIA muscle fibers increased by 23% (41). In contrast, our study integrated PUFA into a more comprehensive dietary score and found that the prevalence of sarcopenia increased by 16% for every one standard deviation increase in aMed. For DASH diet, KUCZMARSKI’s team observed the improvement of grip strength and other representative muscle strength indicators caused by following DASH diet (43). Our study verified the promoting effect of DASH diet on muscle mass with a larger population sample size (*n* = 6,709). In terms of DII, our study found that the prevalence of sarcopenia and osteopenia increased significantly by 44 and 8% per SD rise. A meta-analysis including Asia, the Americas and Oceania showed that compared with the group with lower DII, individuals with higher DII had a 16% increased probability of developing sarcopenia (42). The proportion was 44% in our research, which may be related to the difference in the definition of sarcopenia in different articles. For the relationship between DII and osteopenia, the HR value associated with DII and higher fracture risk was 1.56 in elderly men in Hong Kong (44). And in postmenopausal women in another place, the OR value associated with DII and osteopenia risk was 2.06 (45). Our interaction analysis also identified such sex differences. When analyzing the subgroup of osteopenia in the whole population, DII showed a significant correlation with sarcopenia, which aroused our attention to the promotion of osteopenia on sarcopenia.

This promotion comes from many bioactive factors, especially bone factors. Osteocalcin is an important one of them, synthesized and secreted by osteoblasts. It acts as a biochemical index reflecting bone formation (46), which means its level increases when bone resorption increases. After binding with the osteocalcin receptor GPRC6A in muscle (47), it may influence osteopenia by promoting glucose metabolism, lipid metabolism, insulin secretion, expression and exercise-induced IL-6 secretion (48). Among them, the role of IL-6 is of great importance. First, it forms a pre feedback loop with osteocalcin to regulate and increase the secretion of osteocalcin (49). Second, IL-6 also induces the expression of RANKL (the ligand of NF-κB) to increase and OPG to decrease (50), which leads to the further reduction of bone. Finally, IL-6 itself also has the function of promoting glucose uptake and fatty acid oxidation in muscle (51, 52), playing a synergistic role in promoting metabolism with osteocalcin. In addition, sclerostin released by bone and osteoglycin released by bone and muscle can inhibit the proliferation of myoblasts. They further interfere with glucose metabolism by changing insulin secretion and sensitivity (53, 54).

Represented by SIRI and NRI, the chronic inflammatory state plays an important role in the influence of osteopenia on the correlation between DII score and sarcopenia. The neutrophil-to-lymphocyte ratio (NLR) is considered to be an important parameter to evaluate the systemic inflammatory state and infection risk, and the systemic inflammation response index (SIRI) can reflect the balance between inflammatory response and immune state (34, 55). Many cross-sectional and cohort studies have confirmed the association between DII score and inflammatory markers as follows. Common inflammatory markers include CRP, IL-6 and TNF-a (47). Among them, CRP is the most popular indicator in the study of the relationship between diet score and inflammation. Since the half-life of IL-6 is shorter than CRP, CRP is still the best indicator for inflammatory state (56). As an upstream regulator of CRP and IL6 and its stability in frozen biological samples, NFR is regarded as a reliable alternative marker of TNF-a (47). While SIRI has been recognized as a representative indicator reflecting inflammation (57). Therefore, in this study we especially analyzed the mediating effect of NFR and SIRI between DII and total femur BMD and femoral neck BMD, which turned out to be very significant.

For micronutrients, we found that essential vitamins (VC, VD, VK) or minerals (calcium, phosphorus) can reduce the incidence of sarcopenia and sarcopenia alone or in combination. The combination of VD and calcium has always been regarded as an important treatment for muscle and bone loss (18). In addition to assisting calcium absorption, vitamin D is also related to myoblast differentiation and impaired bone metabolism (58). Previous studies on VC have confirmed its outstanding anti-inflammatory and antioxidant capacity, which may be an important common way to affect sarcopenia and osteopenia. The specific mechanism also includes the regulation of NF-κB to interfere with the production of inflammatory factors TNF *α*, IL-6 and CRP (59), while clearing the excessive accumulation of reactive oxygen species (ROS) (60). As a dietary bioactive substance, Vitamin K plays a role in bone metabolism (12). The specific mechanism may be related to promoting autophagy during osteoblast differentiation and mineralization (61), and further reducing oxidative stress to protect osteopenia (62). There are few studies on the relationship between phosphorus and sarcopenia and osteopenia, and phosphorus was found negatively correlated with sarcopenia and osteopenia in our research. The existing studies focus on the phosphorus metabolites in muscle, because phosphoric acid is an important participant in glucose metabolism and lipid metabolism. A negative correlation between phospholipid levels and markers of sarcopenia has been observed (63). At the same time, bone mineral density of lumbar spine and femoral neck has also been confirmed to be positively correlated with phosphorus intake (64), which may be related to its influence on the secretion of fibroblast growth factor 23 (FGF-23) and parathyroid hormone (PTH) (65). The negative correlation in our research aligns with these documented mechanisms.

The advantages of this study include the use of large-scale nationally representative sample data and a comprehensive assessment of the impact of dietary patterns and important micronutrients. It also considered the interaction between sarcopenia and osteopenia through subgroup analysis. There are also some limitations as follows: (1) This study only analyzed the population of NHANES database, so the conclusion of the article has certain limitations in expanding to the global population; (2) Relying on two 24-h dietary recall questionnaires with an interval of 3–10 days cannot reflect long-term dietary habits; (3) When analyzing the interaction between sarcopenia and osteopenia, the cross-sectional nature of large sample data limits the inference of partial causal correlation; and (4) The interaction between sunlight exposure and nutrients was not fully considered in the analysis.

## 5 Conclusion

In conclusion, adherence to healthy dietary patterns especially anti-inflammatory diet, and supplementary essential vitamins (VC, VD, VK) or minerals (calcium, phosphorus) can reduce the incidence of sarcopenia and osteopenia alone or in combination. Inflammatory indices provide possible biological mechanisms for the effects of dietary patterns on musculoskeletal health.

## Data Availability

Publicly available datasets were analyzed in this study. This data can be found at: https://wwwn.cdc.gov/nchs/nhanes/Default.aspx.
