# Supplementary material for: Associations of four important dietary pattern scores, micronutrients with sarcopenia and osteopenia in adults: results from the National Health and Nutrition Examination Survey
Source: Front Nutr. 2025 Jul 23;12:1583795. doi: 10.3389/fnut.2025.1583795 (PMC12325016; doi:10.3389/fnut.2025.1583795)
Supplement: Supplementary file 1 [file Table_1.docx]

**Supplement information for**

**Associations of four important dietary patterns, micronutrients with sarcopenia and osteopenia in adults: Results from the National Health and Nutrition Examination Survey**

Yuan Li^2,#^, Wen Hao^3,4,#^, Wen Gu^1,#^, Yadan Xu^1^, Kexin Li^1^, Yueliang Zhao^1,*^, Qingyun Huang^1,*^

1. State Key Laboratory of Systems Medicine for Cancer, Center for Single-Cell Omics, School of Public Health, Shanghai Jiao Tong University School of Medicine, Shanghai 200025,
2. Department of Orthopaedics, Peace Hospital of Changzhi Medical College, Shanghai 200333, China;
3. Qingdao Municipal Center for Disease Control and Prevention, Qingdao 266000, China;
4. Qingdao Institute of Preventive Medicine, Qingdao 266000, China;

# Yuan Li, Wen Hao and Wen Gu are co-first authors of the article and contribute equally to this work.

To whom correspondence should be addressed:

*****Qingyun Huang, Ph.D.

Professor of School of Public Health, Shanghai Jiao Tong University School of Medicine, Shanghai, China; E-mail: [qingyunhuang.cityu@gmail.com](mailto:qingyunhuang.cityu@gmail.com)

*Yueliang Zhao, Ph.D.

Professor of School of Public Health, Shanghai Jiao Tong University School of Medicine, Shanghai, China; E-mail: ylzhao1@sjtu.edu.cn

**
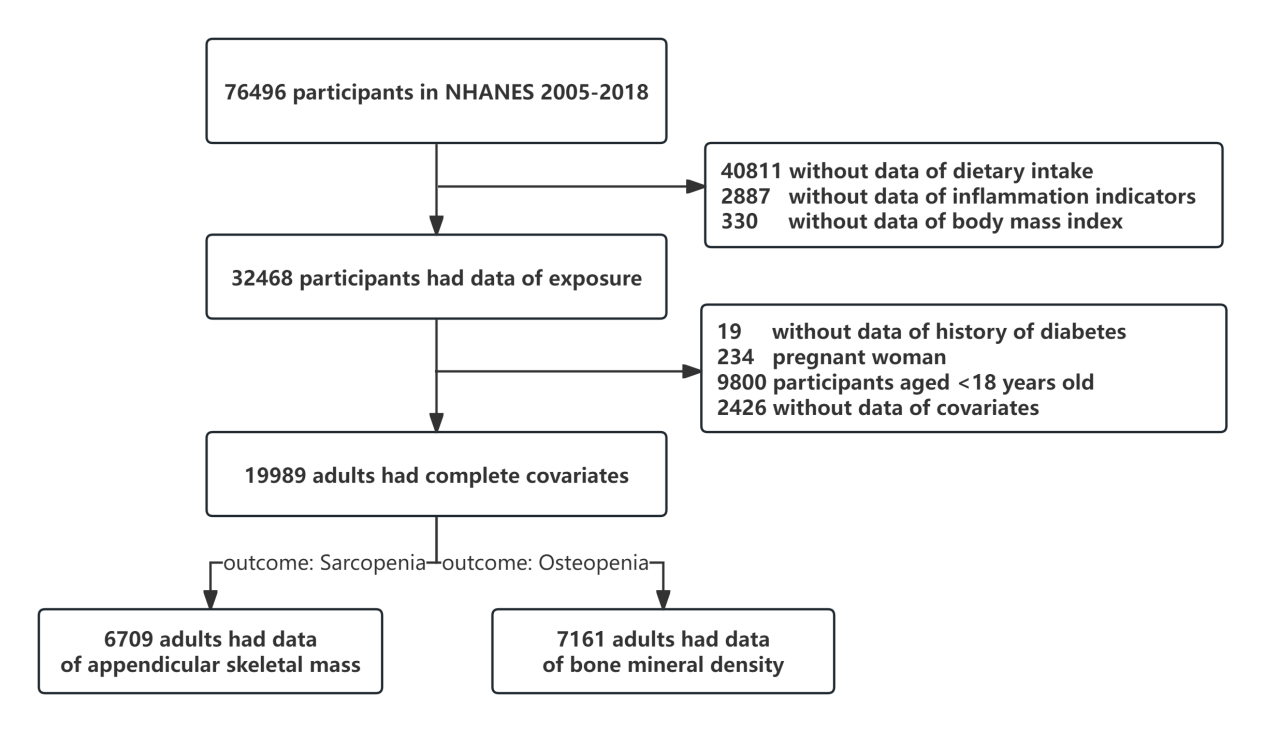
**

**Figure S1.** Flow Chart


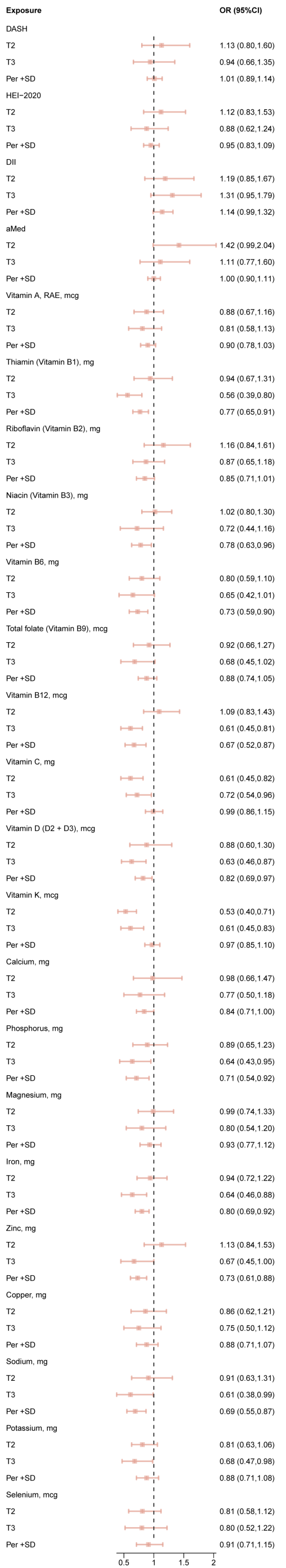


**Figure S2.** Associations of dietary patterns, vitamins, minerals with osteoporosis.

Note: Model : age, sex, race/ethnicity, body mass index, poverty status, education level,history of diabetes, smoking status, alcohol consumption, leisure time physical activity.

**Table S1.** Food Composition of Dietary Pattern Scores.

| Dietary Pattern | Food Composition (maximum score) | Standard for score |
| --- | --- | --- |
| HEI-2020(1) | **Adequacy Components:**  whole fruits, green vegetables, beans, whole grains, dairy foods, total protein foods, seafood, plant proteins, fatty acids.  **Moderation Components:**  refined grains, sodium, added sugars, and saturated fats. | 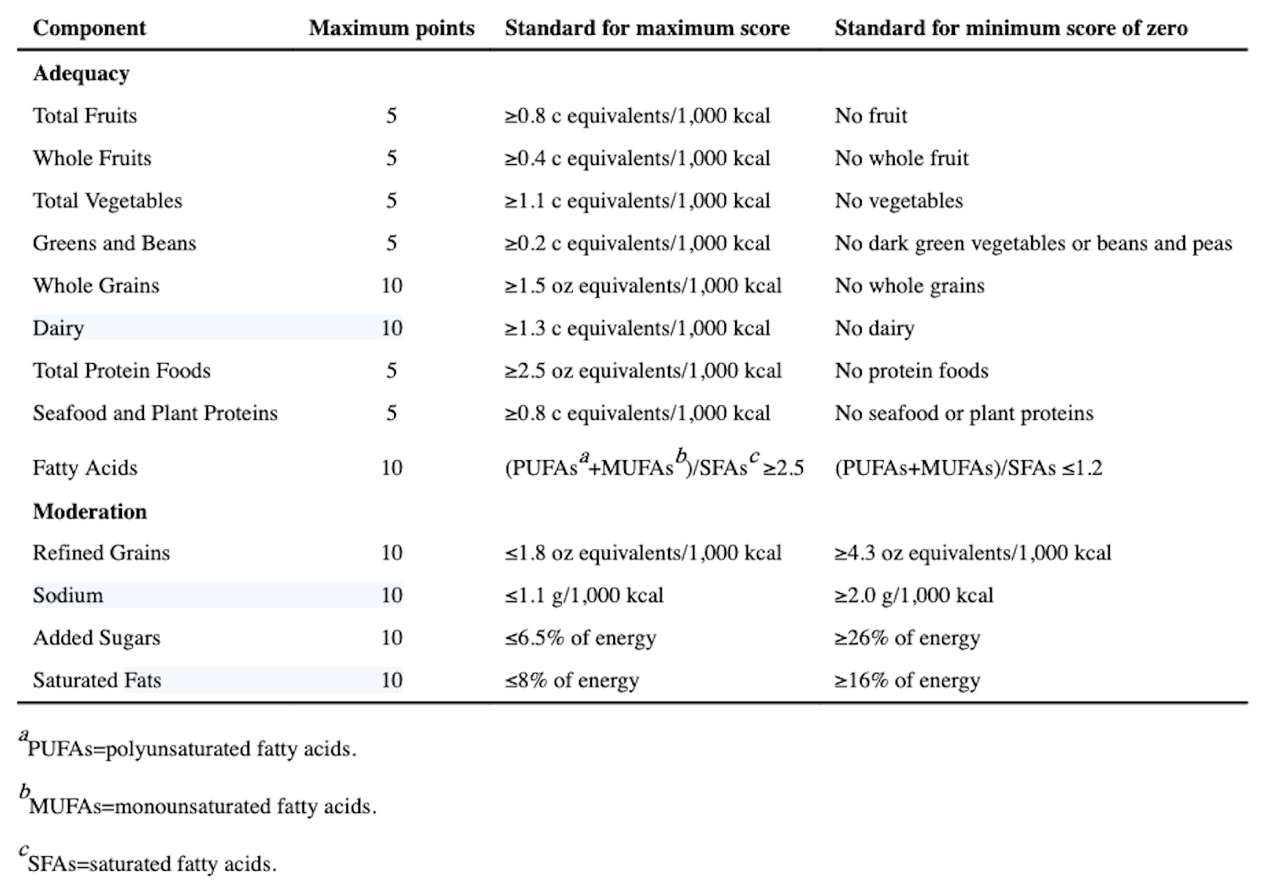 |
| DII(2) | **Pro-inflammatory Components:**  saturated fatty acids, total fat, energy, cholesterol.  **Anti-inflammatory Components:**  polyunsaturated fatty acids, n-3 fatty acids, n-6 fatty acids, monounsaturated fatty acids, fiber, alcohol, vitamin(V) A, VC, VD, VE, magnesium, zinc, selenium, folate, carotene, caffeine.  **Neutral or Context-Dependent Components:**  protein, carbohydrates, niacin, thiamine, VB2, VB6, VB12, iron. | Calculation of the dietary inflammatory index Calculation of the DII is based on dietary intake data that are then linked to the regionally representative world database that provided a robust estimate of a mean and standard deviation for each parameter. These then become the multipliers to express an individual’s exposure relative to the ‘standard global mean’ as a Z-score. This is achieved by subtracting the ‘standard mean’ from the amount reported and dividing this value by its standard deviation. To minimize the effect of ‘right skewing’, this value is converted to a percentile score. To achieve a symmetrical distribution with values centred on 0 (null) and bounded between -1 (maximally anti-inflammatory) and +1 (maximally pro-inflammatory), each percentile score is doubled and then ‘1’ is subtracted. The centred percentile value for each food parameter is then multiplied by its respective ‘overall food parameter-specific inflammatory effect score’ to obtain the ‘food parameter-specific DII score’. Finally, all of the ‘food parameter-specific DII scores’ are summed to create the ‘overall DII score’ for an individual. This approach both ‘anchors’ the individual’s exposure to a robust range of dietary patterns in a variety of cultural traditions and obviates completely the problem of non-comparability of units because the Z-scores and percentiles are independent of the units of measurement. |
| aMed(3) | **Beneficial dietary components:**  vegetables, legumes, fruits, nuts, whole grains, fish,  **Detrimental dietary components:**  meats, red and processed alcohol, and a monounsaturated/saturated fat ratio | Altogether, nine food groups were constructed as index dietary components, namely vegetables, fruits and nuts, cereals, legumes, dairy products, fish and seafood, meat, alcohol, and monounsaturated-to-saturated fat (M/S) ratio. For dietary components that are presumed to be beneficial (i.e., vegetables, fruits and nuts, cereals, legumes, fish and seafood, and a high M/S ratio), we scored a woman that consumed below the median level of the entire cohort as “0” and a woman that consumed at or above the cohort median as 1. For dietary components that are presumed to be less beneficial (i.e., dairy and meat products), a consumption level below the cohort median was given a score of 1 whereas a consumption level at or above the cohort median was given a score of 0. A moderate level of alcohol consumption (5-25 g/day) was scored 1, or 0 otherwise. Scores on all nine components were then summed up as a proxy for adherence to MDP, with the value 0 as the minimal and 9 as the maximal adherence. |
| DASH(4) | total fruit, total vegetables, whole grains, total dairy products, nuts& seeds&legumes, meat/meat equivalents, added sugar, alcoholic beverages, saturated fats. | Using the DASH diet index, participants received one point for consuming the minimum number of servings of select food groups as below. If participants did not consume the minimum number of servings, they received a zero for that individual component. The total index score ranges from zero to nine points, and a higher score indicates better adherence to the DASH diet.  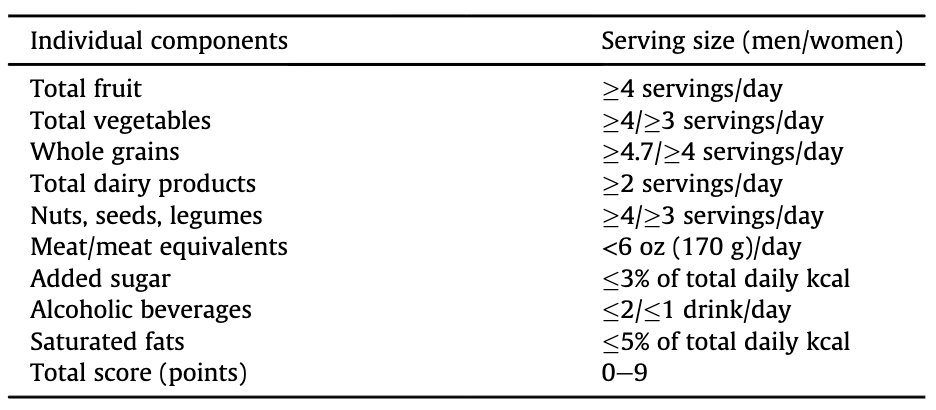 |

Note: All scores were calculated by R package Dietaryindex, details can be found in references(5).

Reference:

1. Sm KS, Te P, Af S, Si K, Jl L, Ja T, et al. Update of the healthy eating index: HEI-2015. J Acad Nutr Diet [Internet]. 2018 Sep [cited 2024 Sep 7];118(9). Available from: http://pubmed-ncbi-nlm-nih-gov-s.webvpn.njmu.edu.cn:8118/30146071/

2. N S, Se S, Tg H, Jr H, Jr H. Designing and developing a literature-derived, population-based dietary inflammatory index. Public Health Nutr [Internet]. 2014 Aug [cited 2025 Apr 25];17(8). Available from: http://pubmed-ncbi-nlm-nih-gov-s.webvpn.njmu.edu.cn:8118/23941862/

3. W Y, M L, R C, Cm H, F F, S S. Mediterranean diet and depression: a population-based cohort study. Int J Behav Nutr Phys Act [Internet]. 2021 Nov 27 [cited 2024 Sep 8];18(1). Available from: http://pubmed-ncbi-nlm-nih-gov-s.webvpn.njmu.edu.cn:8118/34838037/

4. Cc F, Ll K, Ac E. Visceral adiposity index and its association with dietary approaches to stop hypertension (DASH) diet scores among older adults: national health and nutrition examination surveys 2011-2014. Clin nutr (Edinb Scotl) [Internet]. 2021 Jun [cited 2024 Sep 8];40(6). Available from: http://pubmed-ncbi-nlm-nih-gov-s.webvpn.njmu.edu.cn:8118/33640204/

5. Jj Z, Ra H, Al D, Mm L, L B, D L, et al. Dietaryindex: a user-friendly and versatile R package for standardizing dietary pattern analysis in epidemiological and clinical studies. Am J Clin Nutr [Internet]. 2024 Nov [cited 2025 Apr 25];120(5). Available from: http://pubmed-ncbi-nlm-nih-gov-s.webvpn.njmu.edu.cn:8118/39182618/

**Table S2.** Baseline dietary patterns and micro-nutrients of participants in the NHANES.

| **Characteristics** | **Sarcopenia** | | | **Osteopenia** | | |
| --- | --- | --- | --- | --- | --- | --- |
|  | **Yes (N = 738)** | **No (N = 5971)** | **P-value** | **Yes (N = 2947)** | **No (N = 4214)** | **P-value** |
| **Dietary patterns and micro-nutrients, Mean ± SD** | | | | | | |
| **Dietary patterns** | | | | | | |
| HEI-2020 | 50.9 ± 12.8 | 50.4 ± 12.0 | 0.335 | 52.9 ±12.0 | 50.8 ± 11.6 | < 0.001 |
| DII | 1.1 ± 1.8 | 1.0 ± 1.7 | 0.186 | 1.2 ± 1.7 | 1.1 ± 1.7 | 0.227 |
| DASH | 3.6 ± 1.1 | 3.5 ± 1.1 | 0.001 | 3.7 ± 1.2 | 3.6 ±1.1 | < 0.001 |
| aMed | 5.6 ± 1.1 | 5.5 ± 1.0 | 0.185 | 5.8 ± 1.0 | 5.6 ± 1.0 | < 0.001 |
| **Micro-nutrients** | | | | | | |
| Vitamin A, RAE, mcg | 568.7 ± 415.1 | 598.7 ± 466.6 | 0.095 | 627.7 ± 482.1 | 606.8 ± 456.2 | 0.063 |
| Thiamin (Vitamin B1), mg | 1.6 ± 0.8 | 1.6 ± 0.8 | 0.488 | 1.6 ± 0.8 | 1.6 ± 0.8 | < 0.001 |
| Riboflavin (Vitamin B2), mg | 1.9 ± 1.0 | 2.1 ± 1.2 | < 0.001 | 2.0 ± 1.0 | 2.1 ± 1.1 | 0.001 |
| Niacin (Vitamin B3), mg | 24.9 ± 12.4 | 26.9 ± 13.9 | < 0.001 | 23.7 ± 11.9 | 25.8 ± 12.6 | < 0.001 |
| Vitamin B6, mg | 2.0 ± 1.3 | 2.2 ± 1.4 | 0.001 | 2.0 ± 1.2 | 2.1 ± 1.2 | 0.001 |
| Total folate (Vitamin B9), mcg | 416.7 ± 233.9 | 408.3 ± 228.2 | 0.342 | 389.8 ± 206.3 | 408.3 ± 213.9 | < 0.001 |
| Vitamin B12, mcg | 4.8 ± 4.1 | 5.0 ± 4.1 | 0.207 | 4.9 ± 4.3 | 5.2 ± 4.8 | 0.004 |
| Vitamin C, mg | 80.2 ± 76.7 | 82.5 ± 78.3 | 0.454 | 86.2 ± 77.1 | 85.9 ±78.2 | 0.879 |
| Vitamin D (D2 + D3), mcg | 4.4 ± 4.5 | 4.6 ±4.7 | 0.268 | 4.6 ± 4.3 | 4.8 ± 4.4 | 0.207 |
| Vitamin K, mcg | 117.7 ± 130.7 | 121.3 ± 162.0 | 0.567 | 103.2 ± 120.4 | 101.2 ± 116.6 | 0.485 |
| Calcium, mg | 904.7 ± 482.1 | 962.5 ± 517.8 | 0.004 | 892.1 ± 476.8 | 959.5 ± 510.9 | < 0.001 |
| Phosphorus, mg | 1311.3 ± 559.7 | 1406.3 ± 605.4 | < 0.001 | 1273.2 ± 528.8 | 1379.8 ± 571.1 | < 0.001 |
| Magnesium, mg | 290.5 ± 149.3 | 303.0 ± 137.4 | 0.021 | 286.5 ± 121.0 | 297.6 ± 126.2 | < 0.001 |
| Iron, mg | 14.6 ± 7.9 | 14.8 ± 7.7 | 0.585 | 14.6 ± 7.5 | 15.4 ± 7.8 | < 0.001 |
| Zinc, mg | 10.8 ± 5.3 | 11.4 ± 6.1 | 0.013 | 11.1 ± 6.8 | 11.7 ± 6.5 | < 0.001 |
| Copper, mg | 1.2 ± 0.7 | 1.2 ± 0.7 | 0.481 | 1.2 ± 0.7 | 1.3 ± 0.7 | 0.015 |
| Sodium, mg | 3497.8 ± 1481.7 | 3622.3 ± 1551.4 | 0.039 | 3190.9 ± 1394.9 | 3519.4 ± 1531.1 | < 0.001 |
| Potassium, mg | 2518.2 ± 1065.6 | 2619.9 ± 1091.9 | 0.017 | 2595.0 ± 1038.4 | 2662.0 ± 1083.5 | 0.008 |
| Selenium, mcg | 115.5 ± 55.3 | 120.2 ± 56.3 | 0.031 | 105.6 ± 50.9 | 115.3 ± 53.0 | < 0.001 |

Note: Descriptive data were shown as mean (SD) while categorical variables were reported as n (%). P-values less than 0.05 (P-value <0.05) were considered significant.

Abbreviation: NHANES=National Health and Nutrition Examination Survey, N=number, SD=standard deviation, HEI= Healthy eating index, DII=Dietary Inflammation Index, aMed=Alternate Mediterranean Diet, DASH=Dietary Approaches to Stop Hypertension.

**Table S3.** Analysis of the association between DII and sarcopenia in the subgroup of osteopenia.

|  | **Sarcopenia** | | | | | |  |
| --- | --- | --- | --- | --- | --- | --- | --- |
|  | **Model 1** | | | **Model 2** | | | **P interaction** |
|  | OR | 95%CI | P-value | OR | 95%CI | P-value |  |
| Osteopenia |  | | |  | | | 0.037 |
| Low DII | **Reference** | | | **Reference** | | |  |
| High DII | 3.07 | (1.21,7.77) | 0.021 | 3.43 | (1.20,9.77) | 0.024 |  |
| per +SD | 1.86 | (1.20,2.88) | 0.009 | 1.89 | (1.20,3.00) | 0.010 |  |
| Non-osteopenia |  |  |  |  |  |  |  |
| Low DII | **Reference** | | | **Reference** | | |  |
| High DII | 3.10 | (0.95,10.10) | 0.059 | 3.18 | (0.83,12.91) | 0.086 |  |
| per +SD | 1.01 | (0.53,1.93) | 0.970 | 0.97 | (0.53,1.79) | 0.916 |  |

Note: Model 1: Adjusted for age, sex, race/ethnicity, body mass index, poverty status, education level, history of diabetes; Model 2 : Model 1 + Smoking status+ Alcohol consumption+ Leisure time physical activity+ Total energy intake. P-values less than 0.05 (P-value < 0.05) were considered significant.

Abbreviation: DII=Dietary Inflammation Index; OR= odd ratio; CI=confidence interval; aMed=Alternate Mediterranean Diet Score; DASH=Dietary Approaches to Stop Hypertension Index.
